# Intermolecular Charge Transfer Induced Sensitization of Yb^3+^ in β-Diketone Coordination Compounds with Excellent Luminescence Efficiency

**DOI:** 10.3390/ijms26146814

**Published:** 2025-07-16

**Authors:** Trofim A. Polikovskiy, Daniil D. Shikin, Vladislav M. Korshunov, Victoria E. Gontcharenko, Mikhail T. Metlin, Nikolay P. Datskevich, Marat M. Islamov, Victor O. Kompanets, Sergey V. Chekalin, Yuriy A. Belousov, Ilya V. Taydakov

**Affiliations:** 1 P. N. Lebedev Physical Institute of the Russian Academy of Sciences, 53 Leninskiy 1. Prospect, 119991 Moscow, Russia; t.polikovskiy@lebedev.ru (T.A.P.); victo.goncharenko@gmail.com (V.E.G.); belousov@gmail.com (Y.A.B.);; 2 Moscow Institute of Physics and Technology, Institutsky Lane 9, 141700 Dolgoprudny, Moscow Region, Russia; 3 Chemistry Department, M.V. Lomonosov Moscow State University, Leninskie Gory Str., Building 1/3, 119991 Moscow, Russia; marat310105@gmail.com; 4 Bauman Moscow State Technical University, 5/1 2-ya Baumanskaya Str., 105005 Moscow, Russia; 5 Faculty of Chemistry, National Research University Higher School of Economics, 20 Miasnitskaya Str., 101000 Moscow, Russia; 6 Institute of Spectroscopy of the Russian Academy of Sciences, 5 Fizicheskaya Ul., 108840 Moscow, Russia

**Keywords:** Lanthanides, ytterbium, coordination compounds, acylpyrazolones, triphenylarsine oxide, charge transfer, ILCT, NIR luminescence

## Abstract

Achieving high quantum yields for Yb^3+^ ion emission in complexes with organic ligands is a challenging task, as most Yb^3+^ complexes with such ligands typically exhibit efficiencies below 3.5%. Our research demonstrates that the introduction of heavy atom-containing ancillary ligands, such as TPPO or TPAO, along with the careful engineering of the main β-diketone ligand, can increase the luminescence efficiency up to 20-fold by the alteration of the energy migration pathway. It is demonstrated that the combination of two distinct organic ligands leads to the blockage of singlet–triplet intersystem crossing (ISC), alongside electronic energy transfer from β-diketone to Yb^3+^ ions through charge transfer states. The synthesized complexes exhibit quantum yields of 6.5% and 7.0% in the solid state, which places them at the top globally among this class of materials with simple non-deuterated and non-fluorinated ligands.

## 1. Introduction

Yb^3+^ coordination complexes represent a highly promising class of near-infrared (NIR) luminophores owing to their unique photophysical properties. Yb^3+^, which exhibits a single narrow-band luminescence centered at ca. 980 nm, has garnered significant attention in various fields. These emitters find extensive use in biomedical applications [1,2]; they are used to create UV to NIR radiation converters and intensifiers for silicon photoreceivers [3], as well as in in luminescent thermometry [4,5], optoelectronics [5], lasers [6], and other high-tech applications [7,8]. Unfortunately, the relatively low luminescence quantum yields (Φ) of Yb^3+^ coordination compounds pose a significant challenge in their practical application. For the majority of known complexes, Φ does not exceed 3.5% [7]. This is attributed to the extremely low energy (10,250 cm^−1^) of the radiative ^2^F_5/2_ level of the ion. It results in the phonon quenching of emission on one hand, and, on the other, this resonance level lies much lower than the triplet state energies of most organic ligands. Only a few complexes have achieved Φ values of several percent [9,10,11,12,13]. Professor Zhang’s group achieved high yields of 10–23% [14] and a record value of 63% for heterometallic Yb-Co complexes with porphyrin ligands [15]. However, such heterometallic compounds are relatively expensive, and their synthesis is complicated [16,17].

Single-metal complexes with β-diketones, pyrazolonates, or aromatic carboxylates are much cheaper and have less complex synthetic methods, but the highest Φ values do not exceed several percent [18]. In most cases, relatively high quantum yields have been achieved only due to specific experimental conditions. In particular, complexes are dissolved in deuterated or fluorinated solvents [12,13]. For instance, Cameron mentioned [18] that the complex, which demonstrated only Φ = 1.2% in non-deuterated CH_2_Cl_2_, exhibited Φ = 6.5% with a fully deuterated ligand. Moreover, Cameron could not register the quantum yield for the powder complex due to its extremely low luminescence intensity (Φ was less than 0.1%). The required experimental conditions significantly limit the real-world application of monometallic Yb^3+^ compounds due to the highly expensive deuteration procedure [19] used to enhance the luminescence efficiency.

Alternatively, recent studies have shown that introducing triphenylphosphine oxide (TPPO) ligands in Yb^3+^ may increase the luminescence efficiency for solutions of complexes [12]. Hasegawa et al. have suggested that the further enhancement of Φ (in Eu^3+^ complexes) might be possible by substituting phosphorus with the heavier arsenic atom in the ancillary (triphenylarsine oxide, TPAO) ligand [20]. This improvement is attributed to the heavy atom effect, which generally enhances the efficiency of luminescence sensitization in the ligand. However, there is strong concentration quenching in the solid state, which limits the efficiency of monometallic Yb^3+^ compounds, so that achieving a Φ value of several percent for powders still remains a significant challenge.

To date, the impact of arsenic atoms on energy transfer processes and luminescence in Yb^3+^ compounds remains unexplored. It is important to note that the triplet state energy of TPAO is relatively high (26,500 cm^−1^) (see Supplementary Materials for details), making it unsuitable as an antenna ligand for Yb^3+^. Nevertheless, as an ancillary ligand, it can significantly influence the primary antenna ligand, as demonstrated in our previous work [21]. It has also been shown by us previously that the problem of the high energy gap between the excited state of the ligand and the emissive level of Ln^3+^ ions can be solved by an intermediate charge transfer (CT) state [22,23,24,25,26].

Lanthanide derivatives of 1-phenyl-3-methyl-4-acyl-pyrazol-5-ones (HQ) [27,28] are known as effective sensitizers of lanthanide luminescence, especially terbium [29,30] and dysprosium [31,32], due to their suitable triplet level energy, especially in the case of an aliphatic substituent at position R3 [28,33,34,35,36]. Moreover, lanthanide complexes with such ligands are known for the presence of CT states [22,37]. Such CT states can “bridge” energy to the Yb^3+ 2^F_5/2_ emissive level [23,38], thus enhancing the luminescence efficiency.

In this study, we synthesized the HQ^tBu^ ligand for the first time, along with Yb^3+^ complexes with this ligand. The potential of using TPAO as an ancillary ligand in Yb^3+^ ion complexes with a ligand from the β-diketone class was shown. We found that the ligand triplet state did not participate in energy migration from the ligand to the ion. However, the presence of the CT state, with energy close to T_1_ (19,200 cm^−1^), allowed for the efficient sensitization of Yb^3+^. The new complexes with TPPO (**Yb-P**) and TPAO (**Yb-As**) show record-high quantum yields upon β-diketone ligand excitation, presumably due to the alternative energy transfer pathway. A comparative analysis with complexes lacking ancillary ligands (**Gd-H**, **Yb-H**, **Yb-D**) and a Tb^3+^ analog (**Tb-P**) elucidates the observed enhancement in the quantum yield.

## 2. Results and Discussion

### 2.1. Synthesis and Crystal Structure

**Figure 1:**
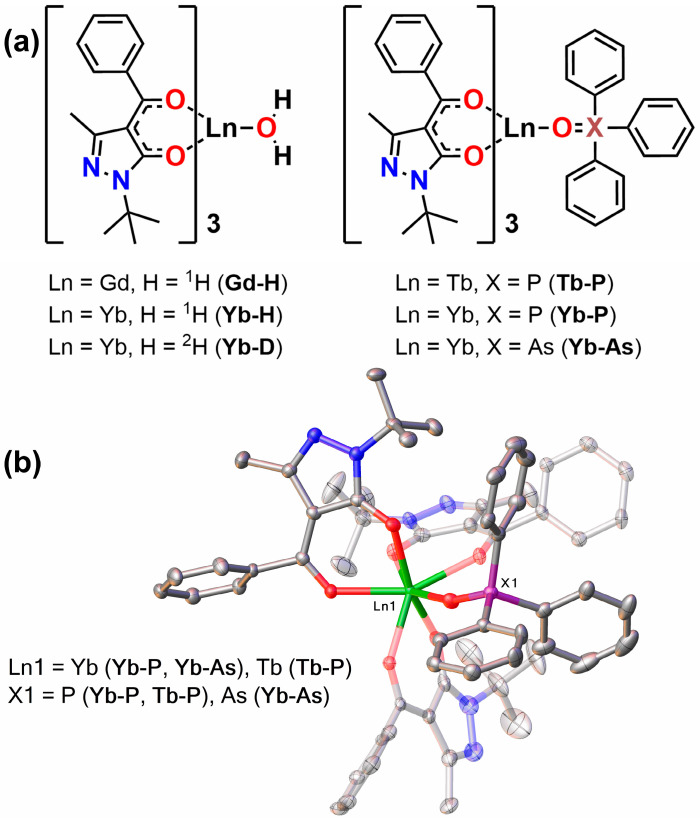
(**a**) Chemical structures of the complexes. (**b**) General view of the molecular structures of **Tb-P, Yb-P**, and **Yb-As**. Thermal ellipsoids of atomic displacement are at 50% probability. Hydrogen atoms are omitted for clarity.

A total of six novel complexes [Ln(Q^tBu^)_3_(H_2_O)] (Ln = Gd and Yb, **Gd-H**, and **Yb-H**, respectively), a deuterated analog of [Yb(Q^tBu^)_3_(H_2_O)] **Yb-D**, [Ln(Q^tBu^)_3_(TPPO)] (Ln = Tb and Yb, **Tb-P**, and **Yb-P**, respectively), and [Yb(Q^tBu^)_3_(TPAO)] (**Yb-As**) (see Figure 1a) were synthesized from the new ligand 4-benzoyl-2-(tert-butyl)-5-methyl-2,4-dihydro-3*H*-pyrazol-3-one (HQ^tBu^). The substances were characterized by elemental analyses, NMR and FTIR spectroscopy, single-crystal X-ray diffraction (SCXRD), and powder X-ray diffraction (PXRD). A detailed description of the synthesis and characterization of the ligand and complexes is given in Section 3.

The slow cooling of the saturated DMF solutions of **Tb-P**, **Yb-P**, and **Yb-As** led to the formation of single crystals suitable for X-ray diffraction. According to the results of SCXRD, **Tb-P**, **Yb-P**, and **Yb-As** are isostructural; thus, further description will be given for the **Yb-As** crystal structure (see Figure 1b).

The complex **Yb-As** crystallizes as a neutral entity with formula [Yb(Q^tBu^)_3_(TPAO)]. The ytterbium ion is coordinated by seven oxygen atoms—six from three bidentate pyrazolone ligands and one from the TPAO ligand—forming a capped octahedral coordination polyhedron.

The average Ln-O (pyrazolone) bond length is slightly elongated in **Tb-P** (2.32 Å) in comparison with Yb complexes (2.28 Å in **Yb-As**, 2.26 Å in **Yb-P**), which is consistent with the expected lanthanide contraction effect.

A comparative analysis of the experimental powder diffraction patterns for **Tb-P**, **Yb-P**, and **Yb-As** with the simulated pattern derived from SCXRD data confirmed the bulk phase purity for all synthesized complexes (see Figures S9–S11, Tables S1 and S2).

### 2.2. Luminescent Properties

**Figure 2:**
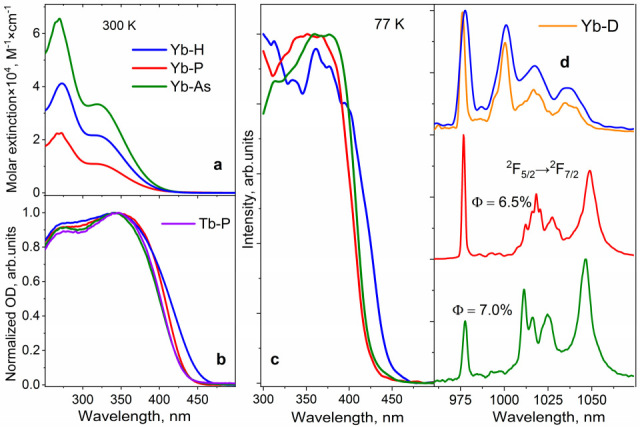
(**a**) UV–Vis spectra, (**b**) diffuse reflectance (DR), and (**c**) luminescence excitation (λ_em_ = 977 nm) spectra. (**d**) PL spectra recorded upon λ_exc_ = 360 nm at 77 K.

The UV–Vis spectra of the compounds in the range of 260 to 400 nm exhibit remarkable similarity, as illustrated in Figure 2a. This suggests that all the observed absorption bands can be attributed to electronic transitions within the β-diketone ligand. The luminescence excitation spectra of all the complexes (see Figure 2c) display similar shapes, with maxima ranging from 350 to 370 nanometers. However, the low-energy edge of the excitation band in the **Yb-P** and **Yb-As** complexes is blue-shifted by 10 nm compared to those complexes that have no additional ligand. Thus, the TPPO and TPAO ligands influence the energy of the low-energy state centered on the diketone ligand. The luminescence spectra (see Figure 2d) of the complexes recorded at 77 K exhibit a narrow band corresponding to the ^2^F_5/2_→^2^F_7/2_ transition of the Yb^3+^ ion in the 950–1100 nm region. Notably, there is no ligand environment fluorescence or phosphorescence, indicating the efficient transfer of electronic excitation energy from the donor ligand to the acceptor ion. The emission band is split into several components, with the most intense peak centered at 976 nm and three weaker peaks observed between 1000 and 1040 nm for **Yb-H**. The Stark sub-bands of the deuterated **Yb-D** complex are better resolved in the spectrum, owing to the suppression of O-H phonon relaxation [31]. The introduction of the ancillary ligands leads to an increase in the number of Stark-splitting components of the emission band, ranging from four to five sub-bands for the **Yb-As** complex and from four to seven sub-bands for the **Yb-P** complex. This observation suggests that the presence of different ancillary ligands leads to variations in the symmetry of the coordination polyhedron.

The overall luminescence quantum yields (Φ) were measured using the absolute method under 365 nm excitation. The measured Φ values, namely 6.5% for **Yb-P** and 7.0% for **Yb-As**, represent exceptional results (see Table 1). The close luminescence lifetimes (τ_obs_) of 31 and 35 μs support the similarity of the Φ values (see Figure S12). It is worth noting that this result is not attributed to the displacement of water molecules from the coordinating sphere, as the Φ of the deuterated complex **Yb-D** without an ancillary ligand (0.4%) is comparable to that of hydrated **Yb-H** (0.3%). Notably, the decay obtained for **Yb-As** dissolved in CDCl_3_ (see Figure S13) exhibits a similar lifetime of 35 μs, indicating no enhancement in luminescence efficiency.

**Figure 3:**
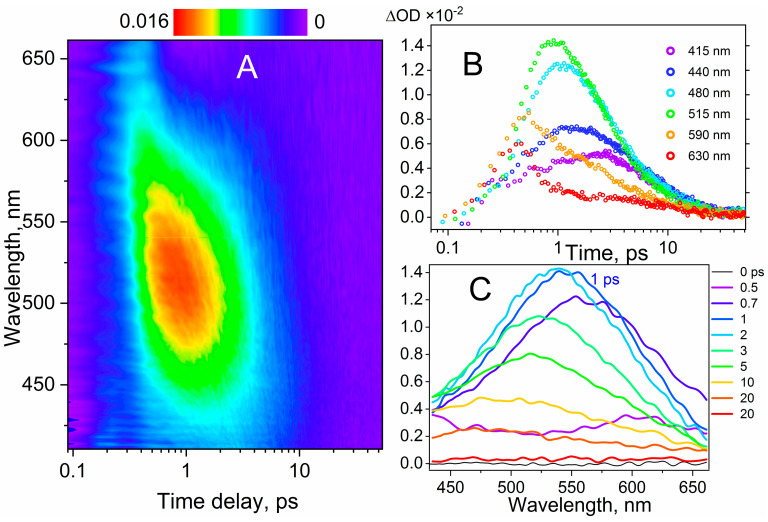
fs-TA counter map plot (**A**), TA kinetic traces at selected wavelengths (**B**), and TA spectra (**C**) measured for [Yb(Q^tBu^)_3_(TPAO)].

To elucidate the nature of the observed phenomenon, we thoroughly investigated the optical properties of the compounds. The energy of the first excited triplet state of the diketone ligand was estimated as 19,200 cm^−1^ (see Figure S14). It is also important to ensure a fast S_1_→T_1_ intersystem crossing (ISC) process. Prof. Malta et al. have previously demonstrated that ISC energy transfer can be enhanced through an intermediate charge transfer state [26]. Notably, the diffuse reflectance (DR) spectra differ from the absorption spectra of the dissolved compounds, with a distinct broad shoulder appearing between 400 and 450 nm (see Figure 2b and Figure 3). We attribute this feature to a charge transfer state.

In order to investigate electronic charge transfer processes within the ligand environment, femtosecond transient absorption (fs-TA) measurements were performed with a pump wavelength of 320 nm. Since the TPAO absorption spectrum appears below 300 nm, it provides excitation through the β-diketone ligand. We observed a positive optical density change, corresponding to excited state absorption (ESA) [21], which was located within 420–660 nm. We also observed two spectral species spawned at a longer spectral region with a maximum at ca. 600 nm and at a short spectral region peaked at 510 nm. The former rapidly decreases within 400–500 fs, suggesting the S_n_→S_m_ energy transition nature of the species. Then, the short-wavelength band intensity rises in the initial period with a lifetime of 0.4 ps, corresponding to the population of the S_1_ state by internal conversion S_n_→S_1_, followed by subsequent relaxation for 6.5 ps. As seen from Figure 3B, all the ligand excited states are fully depopulated within 20 ps, suggesting the absence of long-living T_1_→T_n_ transitions [21,46]. As, generally, β-diketone compounds exhibit T_1_ state lifetimes of ca. 50–200 ps, we conclude that T_1_ does not participate in the electronic energy migration dynamics in our case. Notably, we have already reported similar behaviour for β-diketone compounds previously [46]. This suggests that only ligand singlet excited states are involved in electronic energy migration in the investigated compounds.

**Figure 4:**
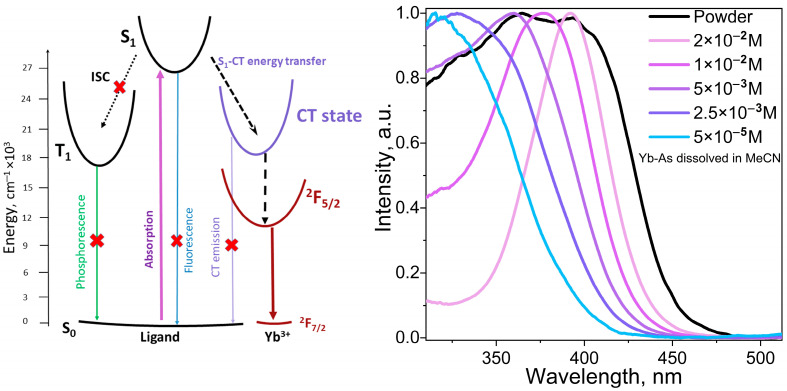
(**left**) Energy level diagram with possible electronic transitions for Yb^3+^ complexes. (**right**) Excitation spectra of **Yb-As** dissolved in MeCN at different concentrations and in solid state.

Due to the high S_1_→Yb^3+ 2^F_5/2_ energy gap (>15,000 cm^−1^), the luminescence quantum yields Φ measured for the dissolved compounds are low (<1%). Seemingly, there is an additional excited state observed for compounds in the solid state, which participates in energy transfer, thus enhancing the luminescence efficiency. We attribute this to a charge transfer state (see Figure 4 left).

Furthermore, luminescence spectra at ambient conditions and in vacuum (10^−4^ Torr) were obtained. Since the ground state of oxygen is triplet, the deaeration of the complex would lead to the enhancement of processes that involve energy transfer through the ligand triplet state. However, we observed no influence of deaeration on the luminescence intensity of complex **Yb-As** (see Supplementary Materials for details). This further suggests that triplet states are not involved in energy relaxation in the investigated complexes.

### 2.3. Charge Transfer Studies

To elucidate the nature of the CT state, DR spectra for the complexes under discussion and **Tb-P** were obtained. Due to high redox potential of Tb^3+^ ions, ligand-to-metal CT (LMCT) cannot occur in Tb^3+^ complexes. As the DR spectra of **Yb-P**, **Yb-As**, and **Tb-P** qualitatively resemble each other, we find that the present state is not of an LMCT nature. Furthermore, we obtained the excitation spectra of **Yb-As** dissolved in MeCN with different concentrations. The CT-related excitation band at 400–500 nm continuously becomes less intense upon dilution. The absorption spectra in solvents with different polarizability were also measured (see Figure S15) with a concentration of ca. 10^−5^ M/L (see Supplementary Materials for details). We observed no presence of CT-related bands. This phenomenon suggests an intermolecular type of CT state, since it is dependent on the average distance between molecules. Thus, we conclude that this state is of an intermolecular CT nature. Presumably, the CT occurs from an electron-rich t-Bu fragment to the diketone moiety between two different molecules.

However, an additional band is observed in the DR spectrum of **Yb-H** in comparison with **Tb-P**, which suggests the presence of an additional LMCT state, namely in **Yb-H**. This is supported by an additional spectral band in the excitation spectrum of **Yb-H** compared to **Yb-P** and **Yb-As**. Generally, it is well known that an LMCT state can suppress the luminescence efficiency, which we observed (Φ of **Yb-P** is significantly lower than Φ of **Yb-P** and **Yb-As**) [22,47,48,49]. Supposedly, ancillary ligands—TPPO and TPAO—suppress the LMCT state, thus reducing the luminescence quenching.

To summarize, the intermolecular CT state is not an intermediate state, as is usually described in the literature [26], but rather sensitizes Yb^3+ 2^F_5/2_ instead of a non-participating ligand T_1_ state. As far as we know, this is the first reported case of such an intricate energy relaxation pathway in β-diketone Yb^3+^ compounds, which may be responsible for the top-rated Φ values of **Yb-P** and **Yb-As**.

## 3. Methods and Materials

### 3.1. Synthesis of 4-Benzoyl-2-(tert-butyl)-5-methyl-2,4-dihydro-3H-pyrazol-3-one

**Scheme 1:**
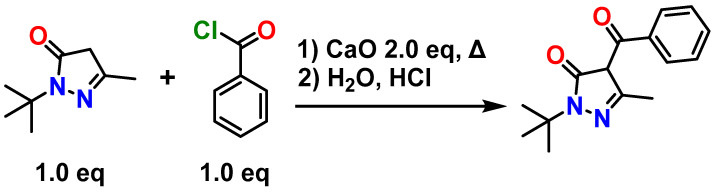
Synthesis of 4-benzoyl-2-(*tert*-butyl)-5-methyl-2,4-dihydro-3*H*-pyrazol-3-one.

In this study, 2-(*tert*-Butyl)-5-methyl-2,4-dihydro-3*H*-pyrazol-3-one was obtained from *tert*-butylhydrazine hydrochloride and ethyl 3-oxobutanoate according to a known synthetic procedure [50]. The ligand HQ^tBu^ was synthesized according to a modified procedure, described earlier by Jensen [51] (Scheme 1). To a solution of 4.83 g (31.3 mmol) of 2-(*tert*-butyl)-5-methyl-2,4-dihydro-3*H*-pyrazol-3-one in 35 mL of dry 1,4-dioxane, 3.52 g (62.8 mmol) of CaO calcined at 1000 °C was added. Then, to the resulting mixture, 3.65 mL (31.3 mmol) of benzoyl chloride was added dropwise with stirring over 30 min. After addition, the yellow mixture was stirred at reflux with the protection of moisture (drying tube) for 4 h and cooled to room temperature. Then, a mixture of 19.5 mL (226.9 mmol) of concentrated HCl and 56.0 mL of H_2_O was added to the reaction mixture. The obtained mixture was concentrated under reduced pressure and diluted with water (50 mL), transferred to a separatory funnel, and extracted with CH_2_Cl_2_ (3 × 20 mL). The combined organic layers were washed with a saturated NaHCO_3_ solution (1 × 35 mL), dried over Na_2_SO_4_, and concentrated to give an orange oil. The resulting oil was purified by column chromatography (silica gel, eluent CH_2_Cl_2_). The yield was 3.52 g (44%) of light yellow crystals. Anal. Calc. for C_15_H_18_N_2_O_2_: C, 69.74; H, 7.02; N, 10.84%. Found: C, 69.85; H, 7.13; N, 10.97%.

^1^H NMR δ (ppm, 400 MHz, CDCl_3_): 7.61–7.42 (m, 5H, Ph), 1.96 (s, 3H, CH_3_), 1.63 (s, 9H, *t*-Bu) (Figure S1).

^13^C NMR δ (ppm, 101 MHz, CDCl_3_): 193.83, 160.80, 145.42, 138.93, 131.45, 128.39, 127.72, 103.24, 59.15, 28.67, 15.81 (Figure S2).

IR ν (cm^−1^): 3434 br, 3058 m, 2977 m, 2932 m, 2872 m, 1603 vs, 1567 vs, 1497 m, 1481 m, 1459 m, 1447 s, 1421 s, 1396 m, 1385 m, 1367 s, 1324 w, 1293 w, 1261 m, 1251 m, 1225 s, 1181 m, 1121 m, 1103 m, 1078 w, 1041 w, 1029 w, 1001 w, 967 m, 931 w, 854 w, 811 m, 799 m, 789 m, 751 m, 702 s, 670 w, 647 w, 613 w, 579 w, 528 w, 486 vw, 407 vw (Figure S3).

### 3.2. Synthesis of Complexes

**Scheme 2:**
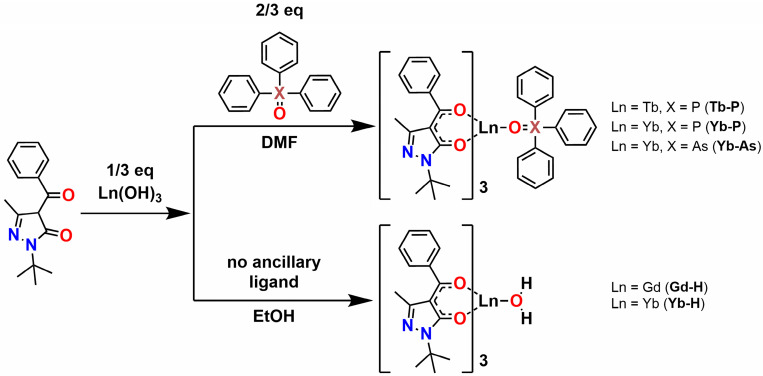
Preparation of complexes.

**Gd-H** and **Yb-H** were synthesized according to the following procedure (Scheme 2): 0.200 mmol of lanthanide chloride or nitrate was dissolved in 1 mL of water in a centrifugation tube. Then, 0.800 mL of a NH_3_ solution (12 M) was added, after which the volume was adjusted to 2 mL with water, and it was centrifuged (8000 rpm, 2 min). The precipitate was carefully washed with water (8 × 2 mL) and added to a solution of 0.600 mmol of HQ^tBu^ in 3 mL of 96% ethanol. The mixture was stirred and refluxed for 5 min and left overnight. The precipitate was separated by vacuum filtration, washed with 2 mL of 96% ethanol, and dried over P_4_O_10_.

Complexes **Tb-P**, **Yb-P**, and **Yb-As** were obtained in a similar way; however, the obtained hydroxide was added to a solution of 0.600 mmol of HQ^tBu^ and 0.400 mmol TPPO or TPAO in 2 mL of DMF. The resulting mixture was also stirred and refluxed for 5 min and left. Precipitated crystals were separated by vacuum filtration and dried over P_4_O_10_ without washing.

The deuterated analog of **Yb-H** (**Yb-D**) was synthesized by keeping 50 mg of the dry complex in a mixture of 200 μL of methanol-*d*_4_ and 200 μL of D_2_O in a desiccator with CaCl_2_ for 24 h [31]. For more complete substitution, the deuteration procedure was repeated twice. Further determination of the properties of **Yb-D**, due to rapid isotopic exchange, was carried out without characterization.

#### 3.2.1. [Gd(Q^tBu^)_3_(H_2_O)] (**Gd-H**)

Yellow solid. Yield 68%. Anal. Calc. for C_45_H_53_N_6_O_7_Gd: C, 57.06; H, 5.64; N, 8.87%. Found: C, 56.92; H, 5.60; N, 8.96%. IR ν (cm^−1^): 3380 br, 3060 m, 2975 m, 2929 m, 2873 m, 1599 vs, 1575 s, 1501 s, 1479 s, 1435 s, 1394 m, 1366 m, 1348 m, 1260 w, 1227 m, 1187 w, 1173 w, 1158 w, 1100 m, 1076 w, 1029 w, 1000 w, 968 m, 924 w, 861 m, 805 m, 766 m, 726 w, 701 m, 666 m, 642 w, 623 m, 581 w, 536 w, 485 w, 431 w (Figure S4).

#### 3.2.2. [Yb(Q^tBu^)_3_(H_2_O)] (**Yb-H**)

Light yellow powder. Yield 57%. Anal. Calc. for C_45_H_53_N_6_O_7_Yb: C, 56.13; H, 5.55; N, 8.73%. Found: C, 55.94; H, 5.62; N, 8.81%. IR ν (cm^−1^): 3399 br, 3061 w, 2978 m, 2931 m, 1631 s, 1596 vs, 1575 s, 1502 s, 1481 vs, 1437 s, 1396 m, 1368 m, 1354 m, 1295 w, 1261 w, 1229 m, 1188 w, 1173 w, 1159 w, 1104 m, 1075 w, 1030 w, 1000 w, 970 m, 931 w, 863 m, 805 w, 789 w, 766 m, 727 w, 703 m, 669 w, 643 w, 625 m, 583 w, 541 w, 491 w, 436 w, 404 w (Figure S5).

#### 3.2.3. [Tb(Q^tBu^)_3_(TPPO)] (**Tb-P**)

Light yellow crystals. Yield 81%. Anal. Calc. for C_63_H_66_PN_6_O_7_Tb: C, 62.58; H, 5.50; N, 6.95%. Found: C, 62.63; H, 5.44; N, 7.05%. IR ν (cm^−1^): 3059 w, 2976 w, 2959 w, 2928 w, 2870 w, 1629 m, 1595 vs, 1575 s, 1527 m, 1500 s, 1479 s, 1461 m, 1436 s, 1393 m, 1368 m, 1350 m, 1260 w, 1231 m, 1164 m, 1122 m, 1099 m, 1074 w, 1029 w, 1000 w, 970 m, 926 vw, 862 w, 805 w, 768 m, 757 w, 726 m, 710 m, 695 m, 665 w, 642 vw, 624 m, 583 vw, 542 s, 491 vw, 467 w, 435 w (Figure S6).

#### 3.2.4. [Yb(Q^tBu^)_3_(TPPO)] (**Yb-P**)

Light yellow crystals. Yield 85%. Anal. Calc. for C_63_H_66_PN_6_O_7_Yb: C, 61.86; H, 5.44; N, 6.87%. Found: C, 61.99; H, 5.63; N, 6.76%. IR ν (cm^−1^): 3059 w, 2976 w, 2961 w, 2929 w, 2871 vw, 1631 m, 1597 vs, 1575 s, 1526 w, 1501 s, 1481 s, 1437 s, 1394 w, 1368 m, 1353 m, 1260 vw, 1231 m, 1168 m, 1122 m, 1102 m, 1074 vw, 1029 vw, 1000 w, 971 m, 926 vw, 862 w, 805 w, 768 m, 758 w, 750 w, 726 m, 708 w, 696 m, 667 w, 642 vw, 625 w, 584 vw, 542 m, 492 vw, 466 vw, 436 w (Figure S7).

#### 3.2.5. [Yb(Q^tBu^)_3_(TPAO)] (**Yb-As**)

Light yellow crystals. Yield 64%. Anal. Calc. for C_63_H_66_AsN_6_O_7_Yb: C, 59.71; H, 5.25; N, 6.63%. Found: C, 59.89; H, 5.37; N, 6.46%. IR ν (cm^−1^): 3059 w, 2976 w, 2960 w, 2928 w, 1631 m, 1597 vs, 1576 s, 1527 w, 1501 s, 1481 s, 1438 s, 1394 w, 1368 m, 1352 m, 1261 vw, 1232 m, 1186 vw, 1176 vw, 1160 vw, 1101 m, 1089 w, 1028 vw, 1000 w, 971 m, 923 w, 909 m, 883 vw, 862 w, 805 w, 768 m, 746 m, 726 w, 696 m, 666 w, 642 vw, 625 w, 583 vw, 538 w, 479 w, 472 w, 463 w, 436 vw (Figure S8).

The crystal phase purity of the obtained samples was confirmed by PXRD data recorded using a Tongda TD3700 diffractometer (Tongda, Dandong, China) (40 kV, 30 mA, CuKα radiation, linear PSD detector) operated in the Bragg–Brentano geometry.

Using Olex2 software package (version 1.5, OlexSys, Regensburg, Germany) [52], the structures were solved with the SHELXT (version SHELXT-2015, Sheldrick, G. M., Göttingen, Germany) [53] structure solution program and refined with the least-squares method against F^2^ in anisotropic approximation for non-hydrogen atoms [54]. All hydrogen atoms were placed in calculated positions and refined within riding model. Main crystallographic details and refinement parameters are listed in Table S1.

The 1H and 13C NMR spectra were recorded on a 400 Mercury Plus Varian (Varian, Palo Alto, CA, USA) spectrometer operating at room temperature.

CHN compositions were estimated with the Elementar Anlysensysteme GmbH “Vario Macro CHN/CHNS” (Elementar Anlysensysteme GmbH, Langenselbold, Germany).

IR spectra were recorded on an IR-Fourier spectrometer, the FT-IR Spectrum One, from PerkinElmer (PerkinElmer, Shelton, CT, USA) in KBr tablets of the 400–4000 cm^–1^ region with a resolution of 0.5 cm^–1^.

Luminescence spectra, luminescence excitation, and luminescence decays were recorded using a Horiba Jobin-Yvon Fluorolog QM-75-22-C spectrofluorimeter (HORIBA, Kyoto, Japan) using a 75 W xenon arc lamp (PowerArc, HORIBA, Kyoto, Japan). A Hamamatsu H10330C (Hamamatsu Photonics, Hamamatsu, Japan) cooled photomultiplier tube sensitive in the NIR region (950–1700 nm) was used as the detector. The spectra were measured at room temperature for powdered samples placed in quartz ampoules with a diameter of 2 mm. For pulsed excitation at 380 nm, a laser system based on a Nd:YAG laser, LQ529B (Solar LS, Minsk, Belarus), with a repetition rate of 20 Hz and 10 ns pulse duration was used. The excitation wavelength was selected with an optical parametric oscillator (OPO), LP604 (Solar LS, Minsk, Belarus), pumped by the second harmonic (532 nm) of the LQ529B. Laser pulses were directed into the Fluorolog spectrofluorimeter’s sample compartment via a custom-designed adaptor. For all optical measurements, the corresponding instrument response functions were taken into account. The experiments were performed in air at atmospheric pressure. Degradation of the optical properties was not observed during the experiments.

The luminescence quantum yields in the visible region were obtained using an absolute method on a home-made setup with a MgO-covered integrating sphere (diameter of 180 mm) and an FD-10G calibrated germanium photodiode detector with a linear amplifier and millivoltmeter; a CW emitting LED (365 nm) was used as an excitation source. Each sample was measured five times under slightly different experimental conditions, and the results were averaged. The average error for the quantum yield measurements was ±15% of the estimated value.

For the transient absorption (TA) experiments, MeOH solutions of the complexes with an optical density of ca. 0.2 were prepared. To avoid photodegradation, solutions were poured into a 1-mm-thick rotating cell. TA spectra were measured on an ultrafast spectrometer based on an optical parametric amplifier (TOPAS, Light Conversion, Vilnius, Lithuania) pumped at 800 nm by a Ti–sapphire femtosecond regenerative amplifier (Spitfire HP, Spectra Physics, Milpitas, CA, USA). The TA measurements were performed with the optical excitation of the ligands in the complexes. For this purpose, laser pulses at 320 nm with a 1 kHz repetition rate and duration of about 100 fs were used. Part of the laser beam from the output of the regenerative amplifier was focused into a water cell to generate a white-light continuum for the broadband probing of the absorption changes. The instrument response function recorded for the MeOH solvent has a characteristic time scale of 300 fs. The threshold irradiation power density was measured by the COHERENT Field Max II (Coherent, Saxonburg, PA, USA).

CCDC 2377318, 2377319, and 2382780 contain the supplementary crystallographic data for this paper. These data can be obtained free of charge via http://www.ccdc.cam.ac.uk (accessed on 14 July 2025) (or from the CCDC, 12 Union Road, Cambridge CB2 1EZ, UK; Fax: +44-1223-336033; E-mail: deposit@ccdc.cam.ac.uk).

## 4. Conclusions

Our investigation of Yb^3+^ coordination compounds has demonstrated notable advancements in luminescence quantum yields (Φ) through the introduction of ancillary ligands—specifically TPPO and TPAO—and the rational design of a diketonate ligand. The ancillary ligands facilitated the achievement of record-high Φ values of 6.5% for **Yb-P** and 7.0% for **Yb-As** in the solid state. This can be attributed to the unusual relaxation pathway, where the ligand triplet state is not involved in energy migration, but, rather, an intermolecular CT state with close to T_1_ energy is observed. The reported quantum yields for Yb^3+^ complexes with non-porphyrin ligands are the highest reported to date. It is important to note that the presented values were obtained for polycrystalline samples of complexes with non-deuterated and non-fluorinated diketonate ligands, which were readily available through a straightforward and cost-effective synthesis process.

## Figures and Tables

**Table 1:** Comparison of the best photophysical results for Yb^3+^ complexes.

Compound	Medium	τ_obs_, μs	Φ, %	Reference
[Yb(Q^tBu^)_3_(TPAO)]	Powder	35	7.0 ^a^	This work
[Yb(Q^tBu^)_3_(TPPO)]		31	6.5 ^a^	
[Yb(tta)_3_(dpso)]·H_2_O	CHCl_3_	–	2.40 ^b^	[1]
[Yb(tta)_3_(dbso)]·H_2_O			1.41 ^b^	
[Yb(tta)_3_(bga)]·H_2_O			1.33 ^b^	
[Yb(tta)_3_(TPPO)]·H_2_O	CHCl_3_	–	1.92 ^b^	[2]
Na[YbL_4_]	Powder	Radiative lifetime (930 μs)	1.3 ^a^	[26]
[Yb(hfaa)_3_(Hind)_2_]	Powder	13.4	0.55 ^a^	[39]
[Yb(hfaa)_3_(pz)_2_]	CHCl_3_	47	3.1 ^b^	[40]
Yb(DBM)_3_ (DPEPO)	Anhydrous DMSO	42.6	0.4 ^b^	[41]
Na_3_[Yb(dpa)_3_]	Tris-HCl	2.23	1.5 ^b^	[42]
[Yb(hfac)_3_DPEPO]	CH_2_Cl_2_	22	2 ^a^	[43]
[NaYb(Lq)_4_]·H_2_O	Powder	19	3.1 ^a^	[44]
[NaYb(LqF)_4_]		20	3.3 ^a^	
[NaYb(LqMe)_4_]		22	3.7 ^a^	
[Yb(L^AB2^)_3_]	Powder CHCl_3_/MeOH	24.8 16.5	1.6 ^a^ 1.1 ^a^	[45]

^a^—measured by absolute method. ^b^—measured by relative method. Q^tBu^—2-(tert-Butyl)-5-methyl-2,4-dihydro-3*H*-pyrazol-3-one; tta—thenoyltrifluoroacetone; dpso—diphenylsulfoxide; dbso—dibenzoylsulfoxide; bga—benzoguinamine; L—dimethyl(4-methylphenylsulfonyl)amidophosphate; hfaa—hexafluoroacetylacetone anion; Hind—indazole; pz—unsubstituted monodentate heterocyclic amine pyrazole; DBM—dibenzoylmethane; DPEPO—bis[2-(diphenylphosphino)phenyl]ether oxide; dpa—dipicolinic acid; hfac—hexafluoroacetylacetonate; Lq, LqF, LqMe—benzoxazole-substituted 8-hydroxyquinolines; L^AB2^—6-(3,5-dimethyl-10-oxo-5,10-dihydro-3H-imidazo[4,5-b]acridin-2-yl)-4-{2-[2-(2-methoxyethoxy)ethoxy]ethoxy}pyridine-2-carboxylic acid.

## Data Availability

The data are available upon reasonable request from the authors. CCDC 2377318, 2377319, and 2382780 contain the supplementary crystallographic data for this paper. These data can be obtained free of charge via http://www.ccdc.cam.ac.uk (accessed on 14 July 2025) (or from the CCDC, 12 Union Road, Cambridge CB2 1EZ, UK; Fax: +44-1223-336033; E-mail: deposit@ccdc.cam.ac.uk).

## Supplementary Materials

The supporting information can be downloaded at: https://www.mdpi.com/article/10.3390/ijms26146814/s1. Reference [55] is cited in the supplementary materials.
