# Supplementary material for: Intermolecular Charge Transfer Induced Sensitization of Yb3+ in β-Diketone Coordination Compounds with Excellent Luminescence Efficiency
Source: Int J Mol Sci. 2025 Jul 16;26(14):6814. doi: 10.3390/ijms26146814 (PMC12295889; doi:10.3390/ijms26146814)
Supplement: Supplementary file 1 [file ijms-26-06814-s001.zip › ijms-3727043-supplementary.pdf]

International Journal of *Molecular Sciences*

MDPI

Supporting Information

Intermolecular Charge Transfer Induced Sensitization of Yb^{3+} in β-Diketone Coordination Compounds with Excellent Luminescence Efficiency

Trofim A. Polikovskiy [1,2], Daniil D. Shikin [3,*], Vladislav M. Korshunov [1,4,*], Victoria E. Gontcharenko [1,5], Mikhail T. Metlin [1,4], Nikolay P. Datskevich [1], Marat M. Islamov [3], Victor O. Kompanets [6], Sergey V. Chekalin [6], Yuriy A. Belousov [1,3] and Ilya V. Taydakov [1]

[1] P. N. Lebedev Physical Institute of the Russian Academy of Sciences, 53 Leninskiy 1. Prospect, 119991 Moscow, Russia; t.polikovskiy@lebedev.ru (T.A.P.); victo.goncharenko@gmail.com (V.E.G.); metlinmt@lebedev.ru (M.T.M.); dac1@yandex.ru (N.P.D.); belousov@gmail.com (Y.A.B.); taidakov@mail.ru (I.V.T.)
[2] Moscow Institute of Physics and Technology, Institutsky Lane 9, 141700 Dolgoprudny, Moscow Region, Russia
[3] Chemistry Department, M.V. Lomonosov Moscow State University, Leninskie Gory Str., Building 1/3, 119991 Moscow, Russia; marat310105@gmail.com
[4] Bauman Moscow State Technical University, 5/1 2-ya Baumanskaya Str., 105005 Moscow, Russia
[5] Faculty of Chemistry, National Research University Higher School of Economics, 20 Miasnitskaya Str., 101000 Moscow, Russia
[6] Institute of Spectroscopy of the Russian Academy of Sciences, 5 Fizicheskaya Ul., 108840 Moscow, Russia; kompanetsvo@isan.troitsk.ru (V.O.K.); schekalin@yandex.ru (S.V.C.)
* Correspondence: d.shikin@lebedev.ru (D.D.S.); korshunovvm@lebedev.ru (V.M.K.)

Experimental 3
Figures 3
Figure S1. ^{1}H NMR spectrum of HQ^{tBu} in $CDCl_3$. 3
Figure S2. ^{13}C NMR spectrum of HQ^{tBu} in $CDCl_3$. 4
Figure S3. IR spectrum of HQ^{tBu} in KBr. 5
Figure S4. IR spectrum of Gd-H in KBr. 6
Figure S5. IR spectrum of Yb-H in KBr. 7
Figure S6. IR spectrum of Tb-P in KBr. 8
Figure S7. IR spectrum of Yb-P in KBr. 9
Figure S8. IR spectrum of Yb-As in KBr. 10
Figure S9. Rietveld PXRD refinement pattern of bulk Tb-P and simulated from single crystal data. 11
Figure S10. Rietveld PXRD refinement pattern of bulk Yb-P and simulated from single crystal data, based on Tb-P structure. 12
Figure S11. Rietveld PXRD refinement pattern of bulk Yb-As and simulated from single crystal data. 13
Figure S12. PL decays for Yb-P and Yb-As recorded upon excitation at 380 nm. 14
Figure S13. PL decay of Yb-As dissolved in $CDCl_3$. 15

Figure S14. Ligand phosphorescence spectrum recorded at 77K........ 16

Figure S15. UV-Vis spectra for Yb-P in toluene, THF and MeCN. 17

Tables 18

Table S1. Main crystallographic details and refinement parameters for structures Tb-P and Yb-As. 18

Table S2. Crystallographic details and Pawley refinement parameters for bulk Tb-P, Yb-P and Yb-As. 19

Notes and references 19

Experimental

Figures

Figure S1. ^{1}H NMR spectrum of HQ^{tBu} in $CDCl_3$.

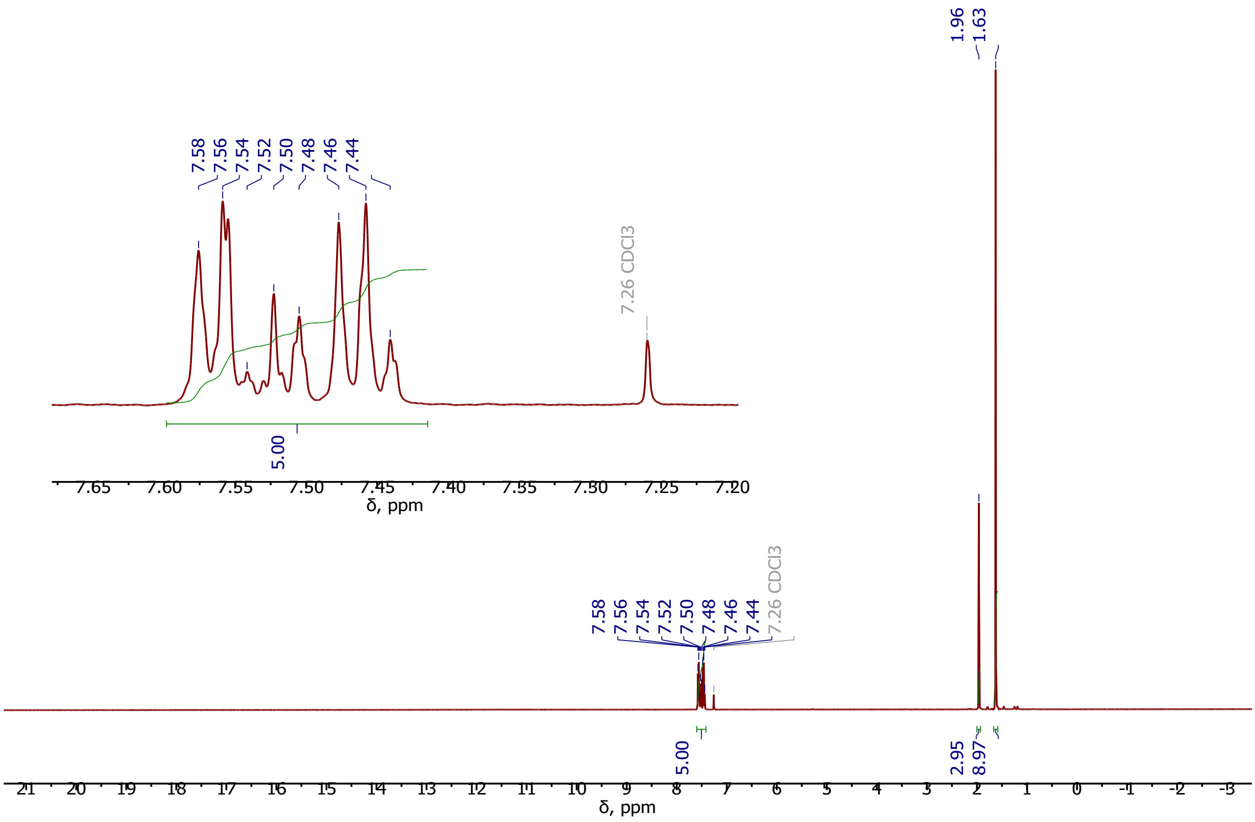

Figure S2. ^{13}C NMR spectrum of HQ^{tBu} in $CDCl_3$.

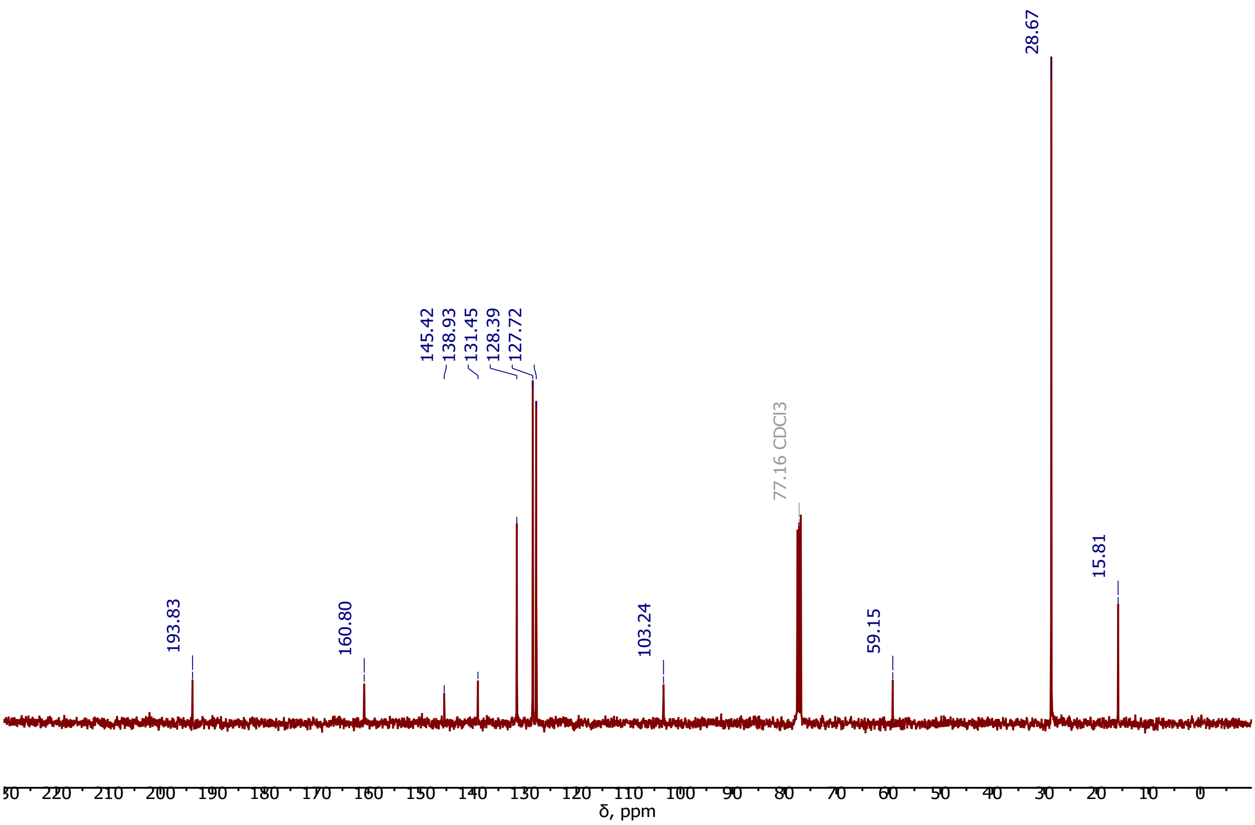

Figure S3. IR spectrum of HQ^{tBu} in KBr.

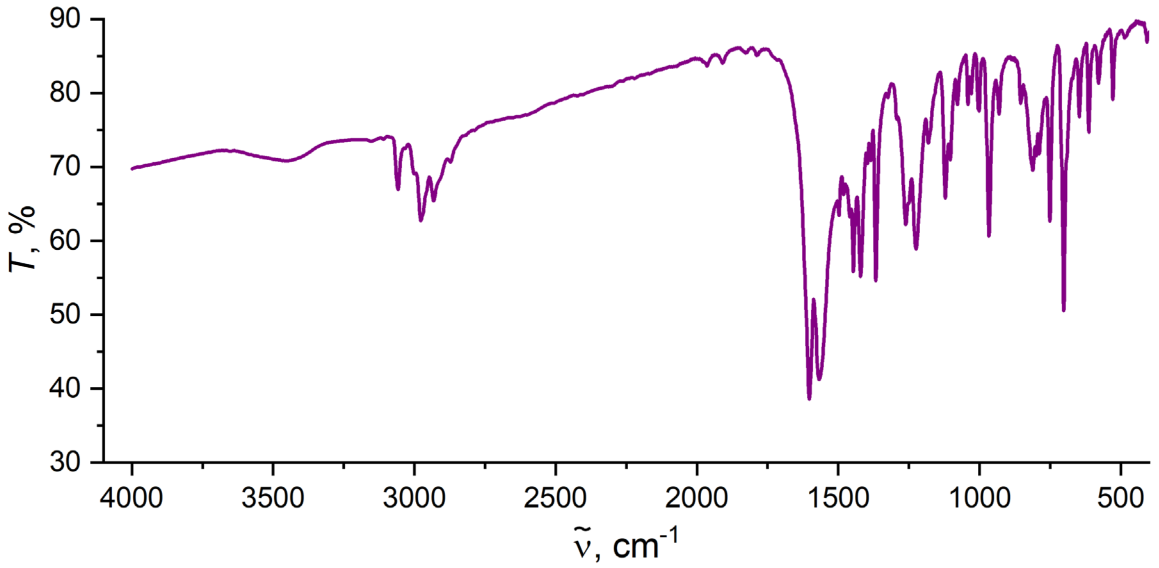

Figure S4. IR spectrum of Gd-H in KBr.

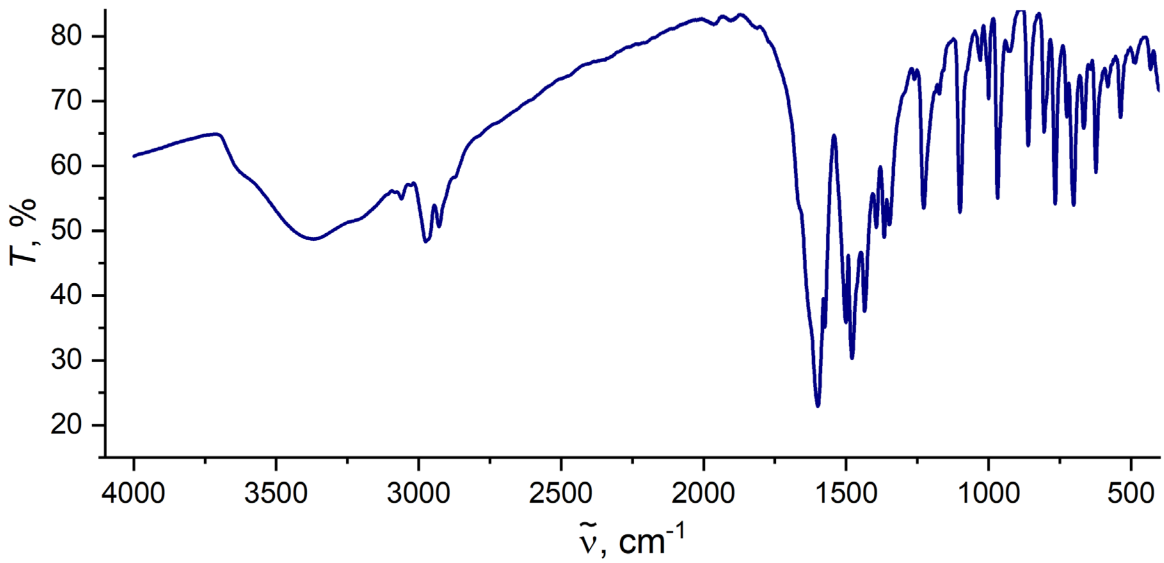

Figure S5. IR spectrum of Yb-H in KBr.

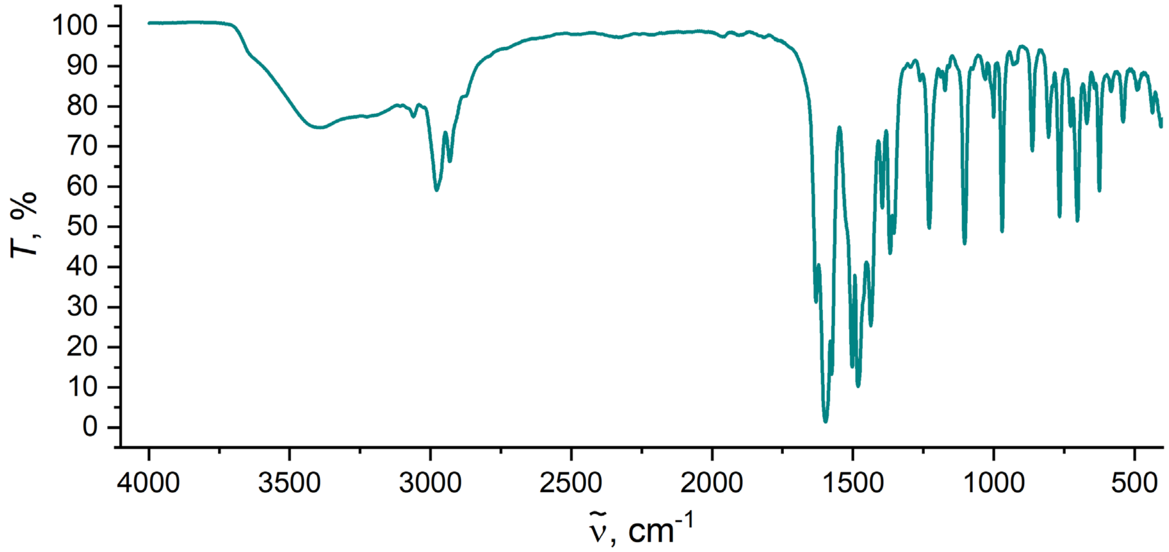

Figure S6. IR spectrum of Tb-P in KBr.

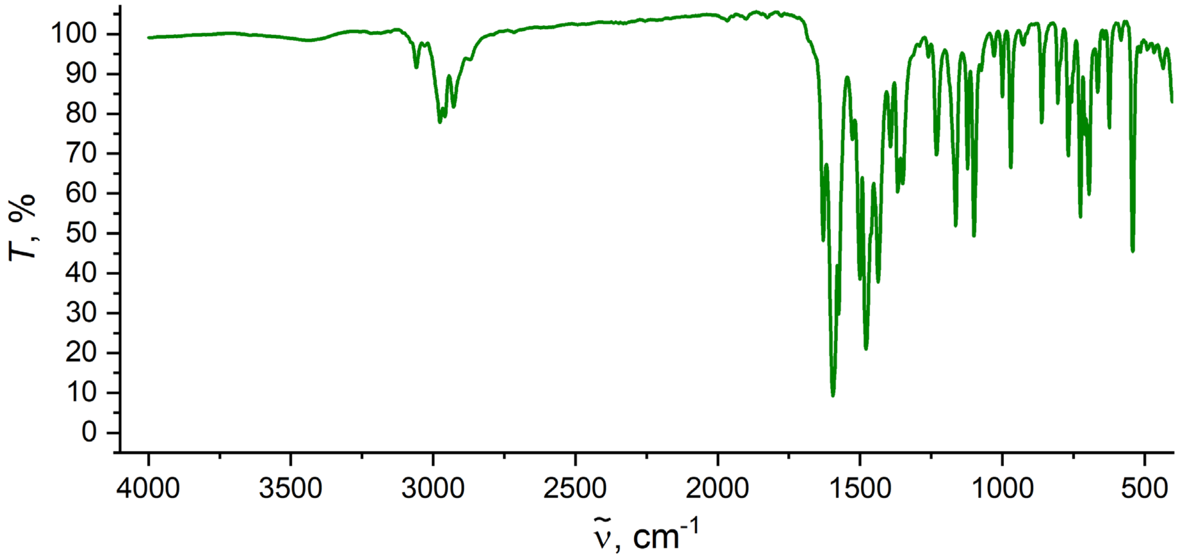

Figure S7. IR spectrum of Yb-P in KBr.

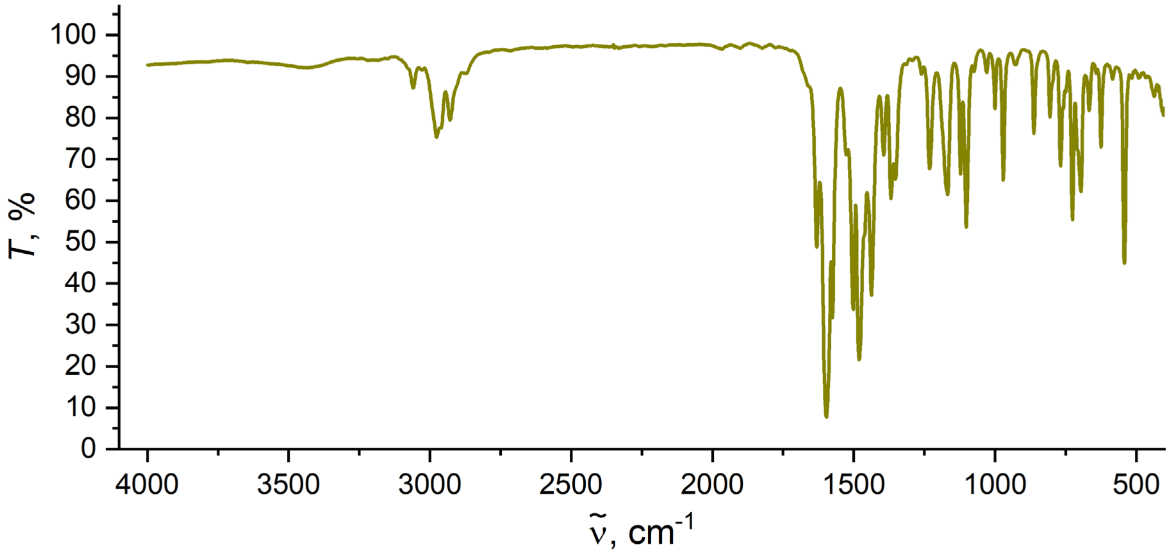

Figure S8. IR spectrum of Yb-As in KBr.

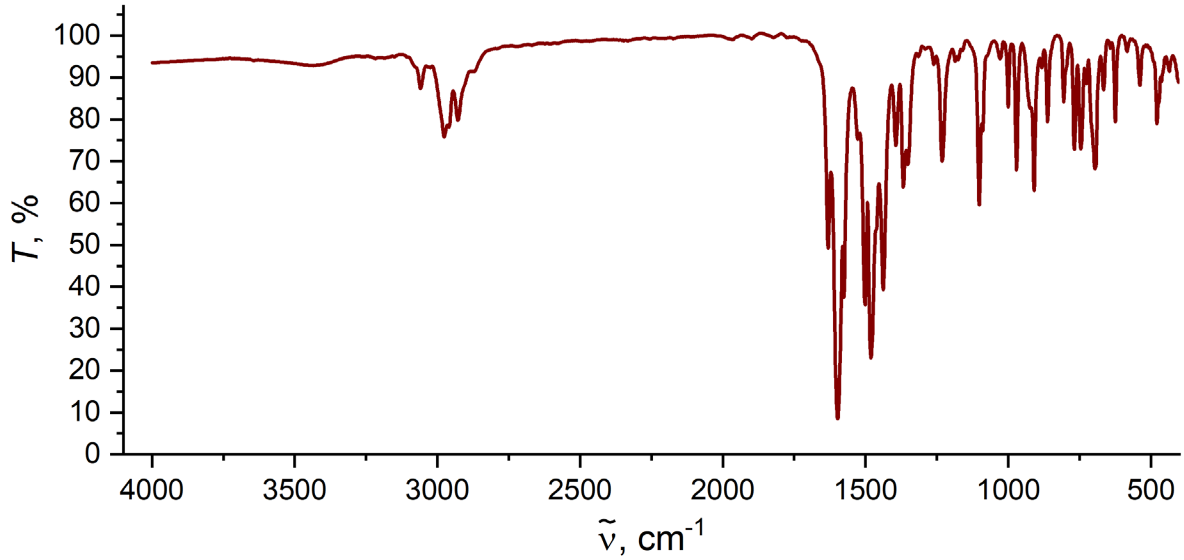

Figure S9. Rietveld PXRD refinement pattern of bulk Tb-P and simulated from single crystal data.

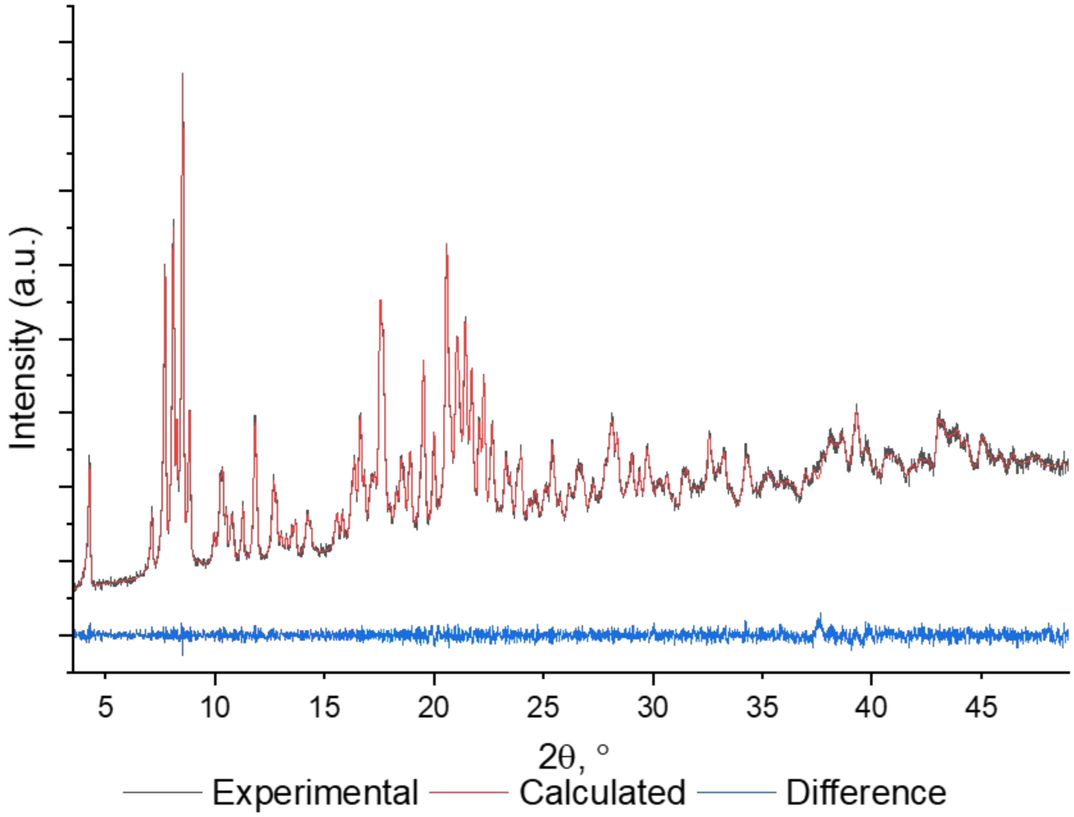

Figure S10. Rietveld PXRD refinement pattern of bulk Yb-P and simulated from single crystal data, based on Tb-P structure.

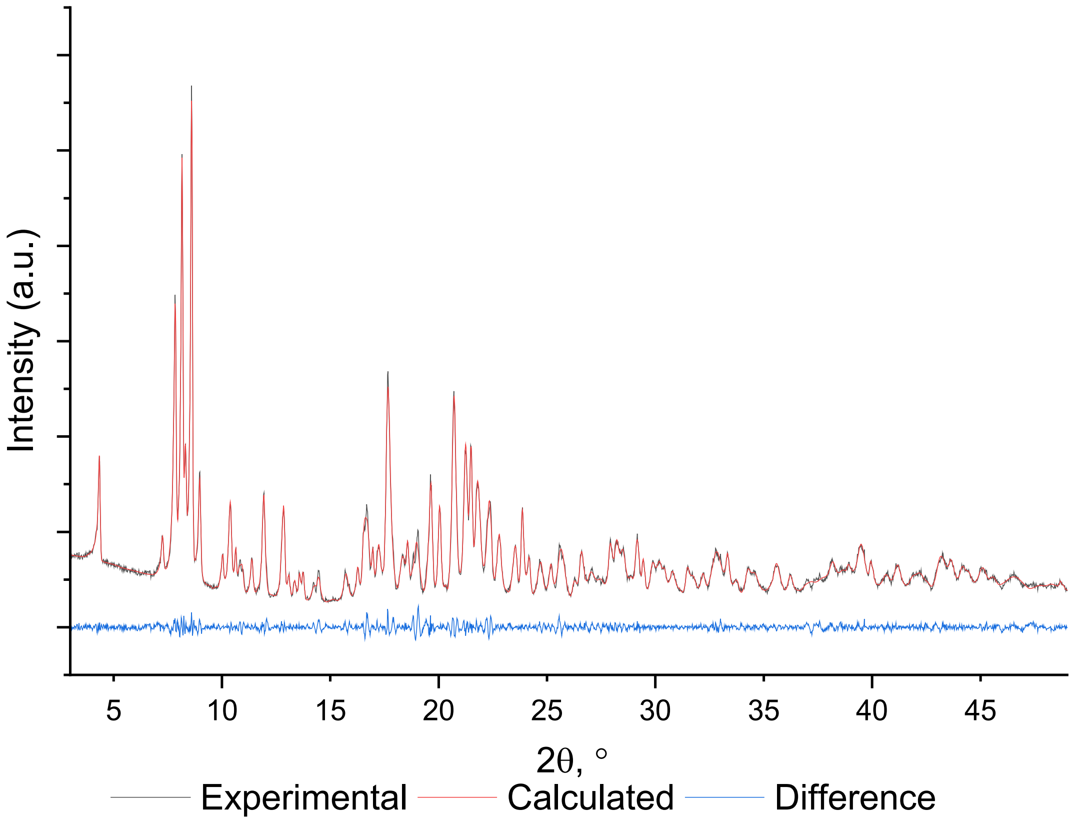

Figure S11. Rietveld PXRD refinement pattern of bulk Yb-As and simulated from single crystal data.

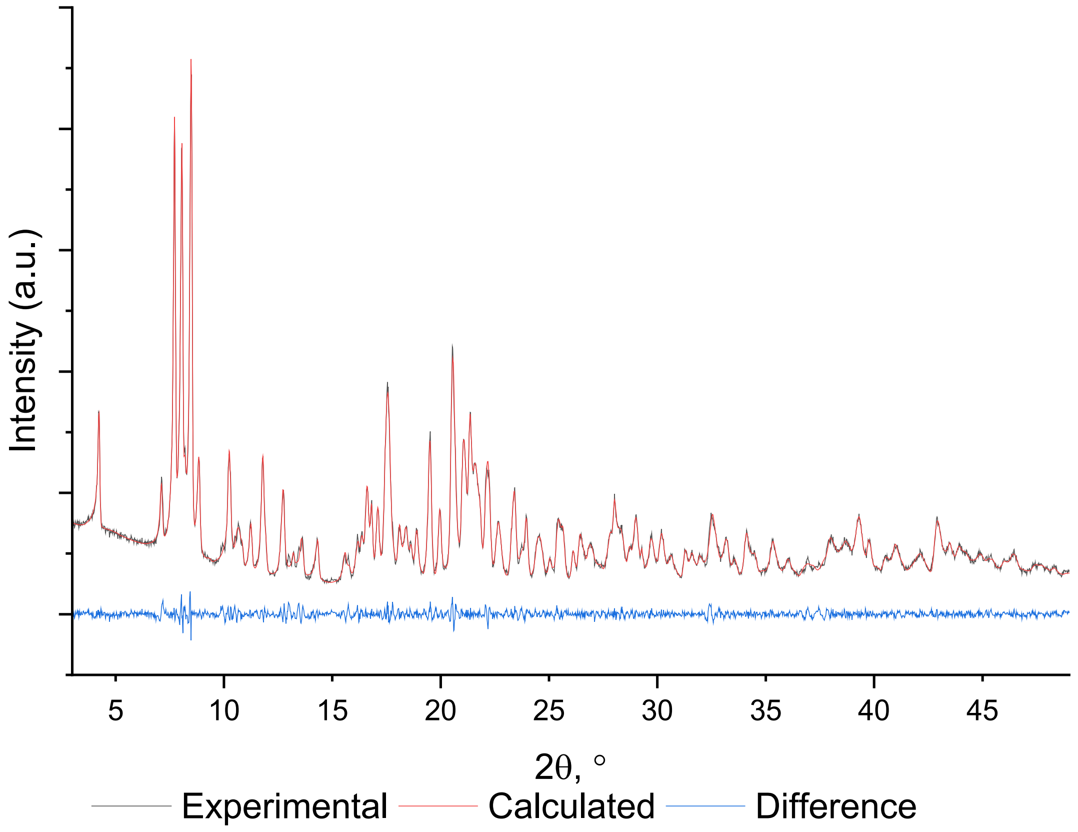

Figure S12. PL decays for Yb-P and Yb-As recorded upon excitation at 380 nm.

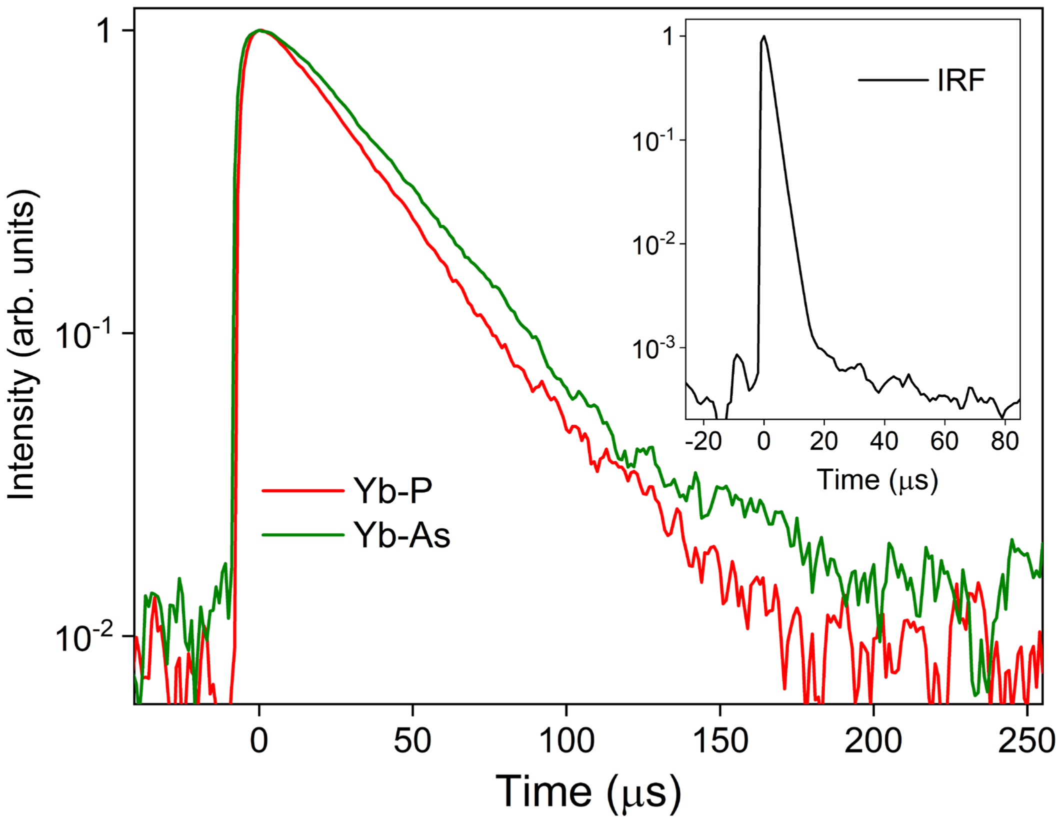

Figure S13. PL decay of Yb-As dissolved in $CDCl_3$.

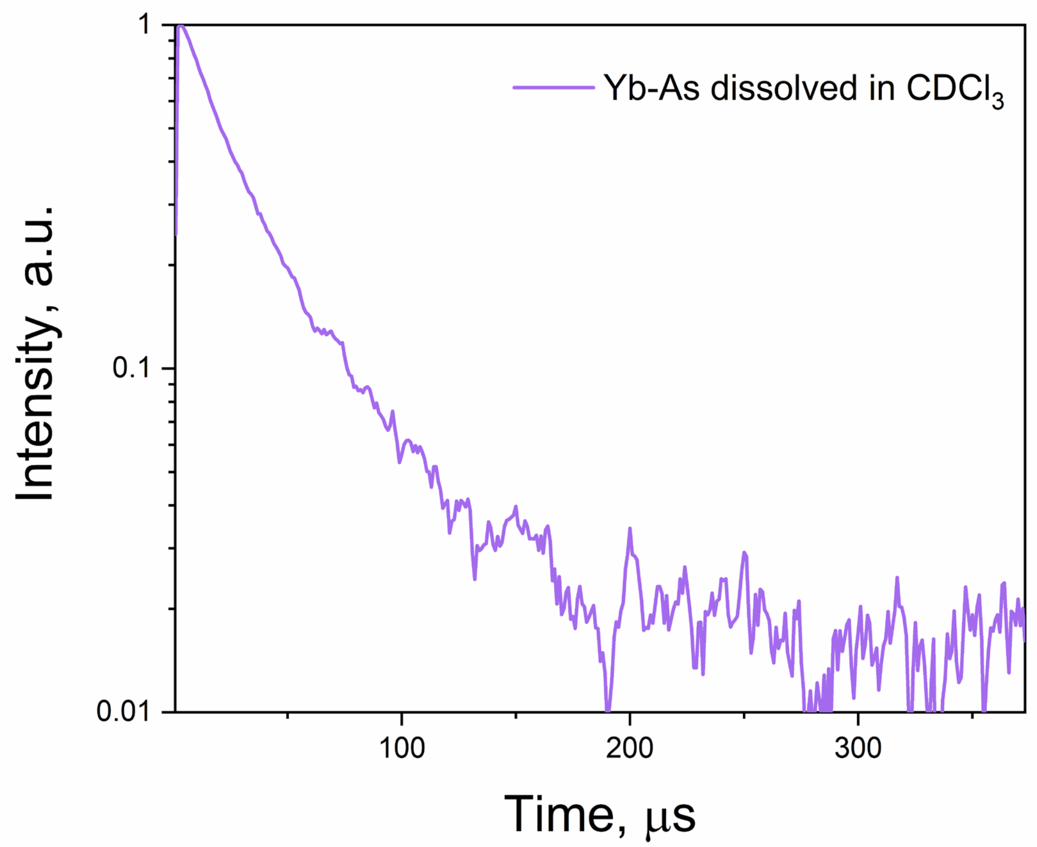

Figure S14. Ligand phosphorescence spectrum recorded at 77K.

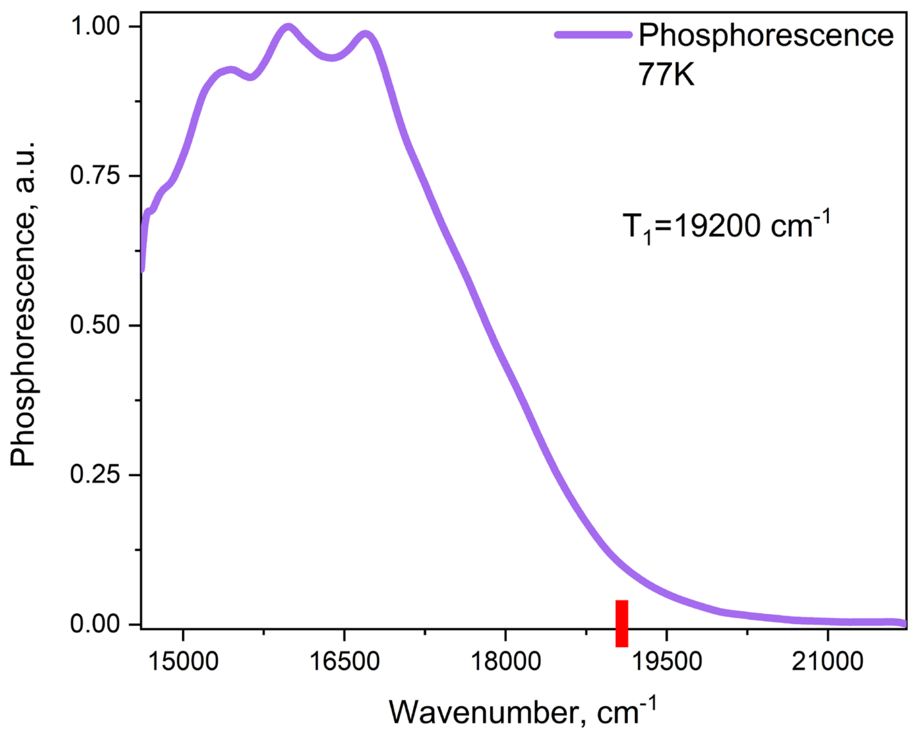

Figure S15. UV-Vis spectra for Yb-P in toluene, THF and MeCN.

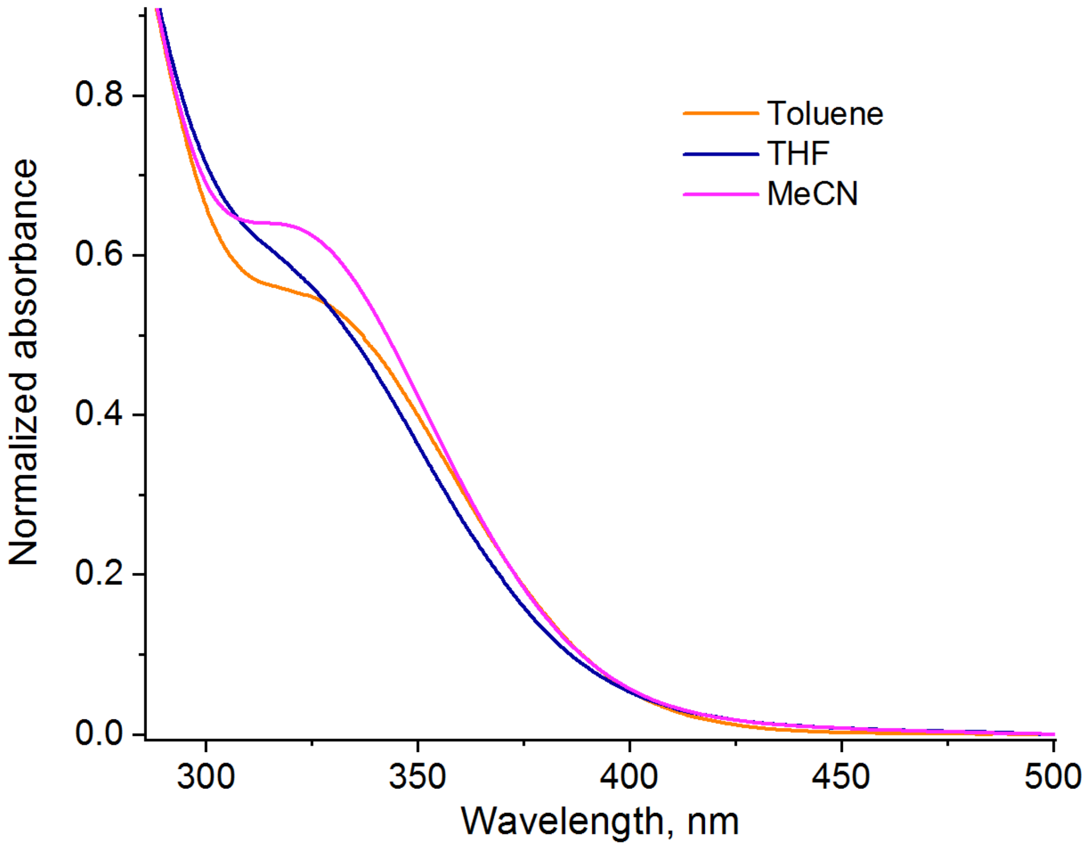

We recorded spectra for solutions with a concentration of approximately 5×10^{-5} M/L. The spectra obtained for Yb-P dissolved in solvents of varying polarity, including low-polarity toluene, THF, and highly polar MeCN, exhibit distinct features in the 300-370 nm region. An increase in solvent polarity corresponds to an increase in the absorption intensity of the band located within this range (see Figure S15). However, the spectra remain consistent in the higher-energy region (<300 nm). Consequently, this band can be attributed to an intramolecular charge transfer (ILCT) state[1].

Tables

Table S1. Main crystallographic details and refinement parameters for structures Tb-P and Yb-As.

Compound	Tb-P	Yb-P	Yb-As
CCDC	2377318	2382780	2377319
Empirical formula	$C_{63}H_{66}PN_6O_7Tb$	$C_{63}H_{66}PN_6O_7Yb$	$C_{63}H_{66}AsN_6O_7Yb$
Formula weight (g·mol^{-1})	1209.10	1223.22	1267.17
T (K)	120(2)	100(2)	100(2)
Crystal system	Triclinic	Triclinic	Triclinic
Space group	*P-1*	*P-1*	*P-1*
a (Å)	11.1347(8)	11.1658(5)	11.1693(6)
b (Å)	12.4351(8)	12.3058(6)	12.3562(6)
c (Å)	21.2813(15)	21.2803(10)	21.3438(10)
α (deg)	81.069(3)	80.606(2)	80.944(2)
β (deg)	76.835(3)	76.466(2)	76.214(2)
γ (deg)	88.761(3)	89.288(2)	88.999(2)
V (Å^3)	2834.1(3)	2803.7(2)	2824.5(2)
Z	2	2	2
D_{calc} (g·cm^{-3})	1.417	1.449	1.490
θ_{min}-θ_{max} (deg)	1.878 – 26.999	1.805 – 29.000	1.669 – 29.000
μ (mm^{-1})	1.335	1.756	2.296
T_{min}/T_{max}	0.5724/0.6478	0.5952/0.6478	0.5111/ 0.6478
Total/Unique reflections number	31122/12311	40857/14884	26912/14838
Reflections with $I > 2\sigma(I)$	10170	13128	12030
R_{int}	0.0687	0.0599	0.0575
GooF	1.042	1.059	1.017
R_1, wR_2 ($I > 2\sigma(I)$)	0.0510, 0.1096	0.0464, 0.0961	0.0529, 0.1082
R_1, wR_2 (all data)	0.0642, 0.1182	0.0542, 0.1001	0.0675, 0.1164

Table S2. Crystallographic details and Pawley refinement parameters for bulk Tb-P, Yb-P and Yb-As.

Compound	Tb-P	Yb-P	Yb-As
a (Å)	11.2305(4)	11.2389(7)	11.2569(9)
b (Å)	12.5923(6)	12.4646(1)	12.5163(11)
c (Å)	21.5749(9)	21.5793(1)	21.6205(17)
α (deg)	81.070(3)	80.974(5)	81.057(6)
β (deg)	76.866(6)	76.920(5)	76.746(7)
γ (deg)	88.398(3)	88.595(6)	88.691(6)
V (Å^3)	2935.20(7)	2907.92(5)	2928.80(4)

Notes and references

1. Zhang, Z.; Zhou, Y.; Li, H.; Gao, T.; Yan, P. Visible Light Sensitized Near-Infrared Luminescence of Ytterbium *via* ILCT States in Quadruple-Stranded Helicates. *Dalton Trans.* **2019**, *48*, 4026–4034, doi:10.1039/C9DT00614A.
